# Supplementary material for: Whole Exome Sequencing as a Diagnostic Tool for Unidentified Muscular Dystrophy in a Vietnamese Family
Source: Diagnostics (Basel). 2020 Sep 24;10(10):741. doi: 10.3390/diagnostics10100741 (PMC7598670; doi:10.3390/diagnostics10100741)
Supplement: Supplementary file 1 [file diagnostics-10-00741-s001.zip › diagnostics-900787-supp-xml-2/Figure S1 and S2 xml.docx]

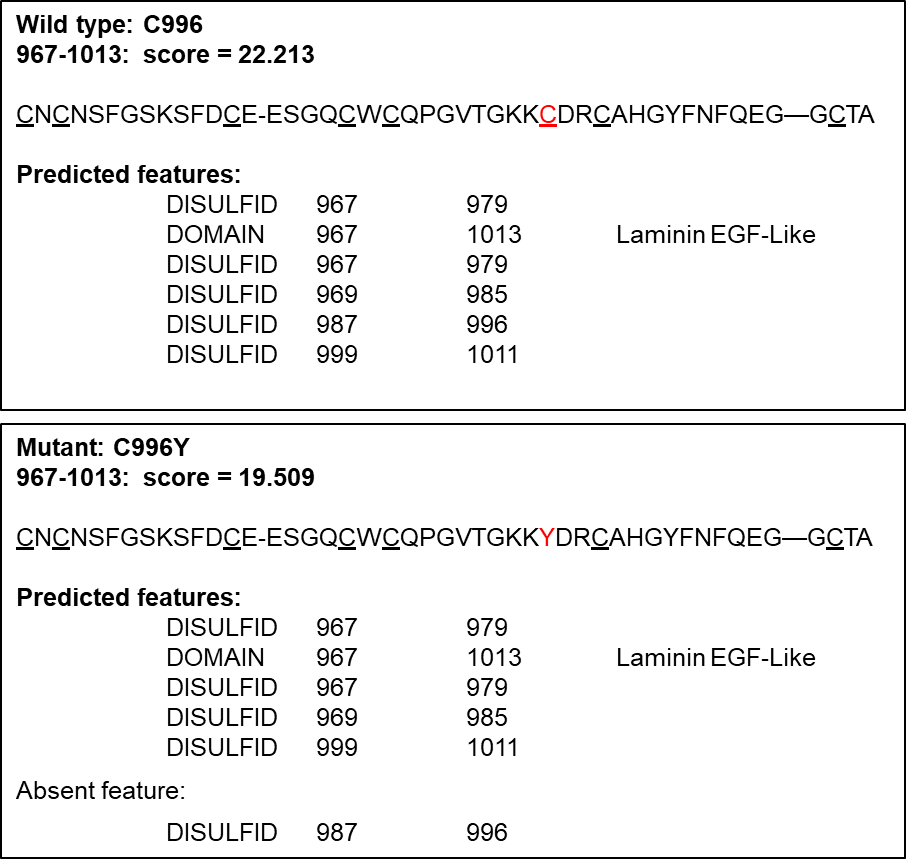


**Figure S1.** ScanProsite predicted disulfide bonds of wild type C996 and mutant form Y996 in the laminin EGF-like domain corresponding to residues 97-1013 of LAMA2.


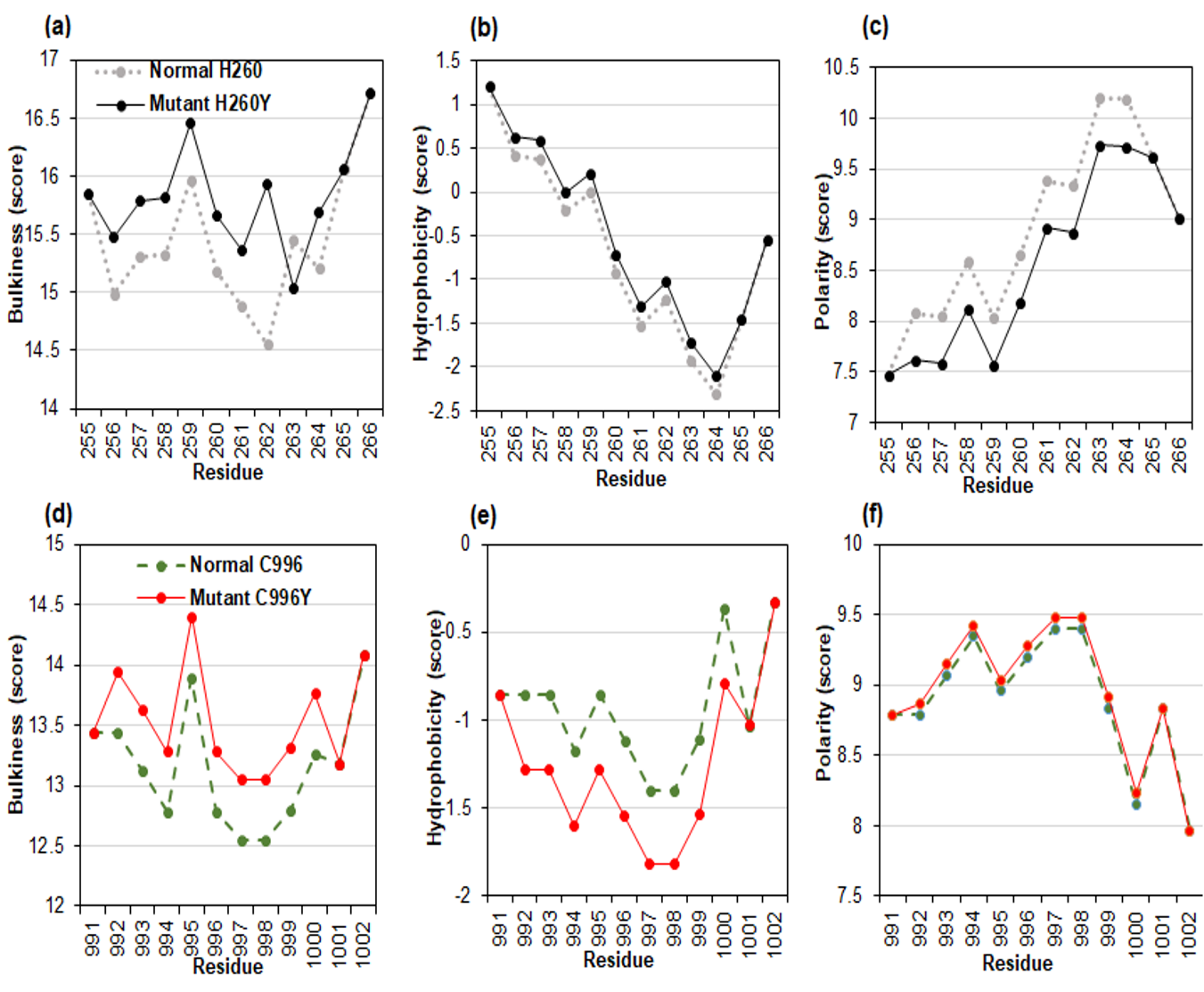


**Figure S2.** Prediction of bulkiness (**a,d**), hydrophobicity (**b,e**), and polarity (**c,f**) of H260Y and C996Y and amino acids nearby using ProtScale tool.
