# Supplementary material for: Whole Exome Sequencing as a Diagnostic Tool for Unidentified Muscular Dystrophy in a Vietnamese Family
Source: Diagnostics (Basel). 2020 Sep 24;10(10):741. doi: 10.3390/diagnostics10100741 (PMC7598670; doi:10.3390/diagnostics10100741)
Supplement: Supplementary file 1 [file diagnostics-10-00741-s001.zip › diagnostics-900787-supp-xml-2/Supplementary Table S1 xml.docx]

**Supplementary Table S1.** Summary of the whole exome sequencing data and filtered procedure.

| **Parameters** | **Patient P1** | **Patient P2** | **Father** | **Mother** |
| --- | --- | --- | --- | --- |
| Total reads | 62,355,218 | 74,719,830 | 76,051,046 | 72,118,676 |
| Total read bases (bp) | 9,255,077,686 | 11,087,424,339 | 11,360,129,884 | 10,750,365,256 |
| Bases with Q > 30 (%) | 93.1 | 93.0 | 94.6 | 94.8 |
| Target regions (bp) | 60,456,963 | 60,456,963 | 60,456,963 | 60,456,963 |
| Average throughput depth of target regions (×) | 153.0 | 183.3 | 187.9 | 177.8 |
| Initial mappable reads  (mapped to human genome) | 62,274,429  (99.8%) | 74,623,904  (99.8%) | 75,993,929 (99.9%) | 72,068,593  (99.9%) |
| Non-redundant reads | 56,824,072  (91.2%) | 67,524,603  (90.4%) | 64,795,013  (85.2%) | 61,738,604  (85.6%) |
| On-target reads | 43,842,098  (77.1%) | 52,987,831  (78.4%) | 44,033,664  (67.9%) | 39,970,727  (64.7%) |
| Mean depth of target regions (×) | 89.2× | 107.7× | 88.6× | 80.9× |
| % Coverage of target regions (>10×) | 98.7 | 98.9 | 98.5 | 98.0 |
| % Coverage of target regions (>20×) | 95.6 | 97.1 | 95.0 | 93.8 |
| % Coverage of target regions (>30×) | 90.0 | 93.7 | 88.8 | 86.6 |
| Number of SNPs | 95,859 | 97,062 | 96,300 | 96,629 |
| Number of Indels | 13,626 | 14,332 | 14,100 | 13,762 |
| Number of SNPs/Indels in genes associated with neuromuscular disorders | 4,622 | 4,614 | 4,511 | 3,405 |
