# Supplementary material for: Whole Exome Sequencing as a Diagnostic Tool for Unidentified Muscular Dystrophy in a Vietnamese Family
Source: Diagnostics (Basel). 2020 Sep 24;10(10):741. doi: 10.3390/diagnostics10100741 (PMC7598670; doi:10.3390/diagnostics10100741)
Supplement: Supplementary file 1 [file diagnostics-10-00741-s001.zip › diagnostics-900787-supp-xml-2/Supplementary Table S3 xml.docx]

**Supplementary Table S3.** Classification of the identified variants according to the American College of Medical Genetics and Genomic recommendations.

| **c.778C>T (p.H260Y): Likely Pathogenic** | | |
| --- | --- | --- |
| 1 Moderate | PM2 | Extremely low frequency  This rare variant is observed in heterozygous state with allele frequency accounting for 4 out of 250,644 in gnomAD_exome and 3 out of 120,950 in ExAC |
| 4 Supports | PP1 | Co-segregation with disease |
|  | PP2 | Missense variant in a gene that has low rate of benign missense variation and in which missense variants are a common mechanism of disease |
|  | PP3 | + PolyPhen-2 and Mutation Taster predicted disease causing.  + A histidine residue at the position 260 was highly complete conservative among distinct species. |
|  | PP4 | Patient’s phenotype or family history is highly specific for a disease with a single genetic etiology |
| **c.2987G>A (p.C996Y): Likely Pathogenic** | | |
| 2 Moderates | PM2 | Absent in 1,000 Genomes Project, Exome Variant Project, Genome Aggregation Database |
|  | PM5 | Novel missense change at an amino residue where a different missense change determined to be pathogenic has been seen before  C996R is pathogenic; this study: C996Y. |
| 4 Supports | PP1 | Co-segregation with disease |
|  | PP2 | Missense variant in a gene that has low rate of benign missense variation and in which missense variants are a common mechanism of disease |
|  | PP3 | + SIFT, PolyPhen-2, and Mutation Taster predicted disease causing.  + A histidine residue at the position 996 was highly complete conservative among distinct species. |
|  | PP4 | Patient’s phenotype or family history is highly specific for a disease with a single genetic etiology |
